# Dasatinib Inhibits Lung Cancer Cell Growth and Patient Derived Tumor Growth in Mice by Targeting LIMK1

**DOI:** 10.3389/fcell.2020.556532

**Published:** 2020-12-04

**Authors:** Man Zhang, Jie Tian, Rui Wang, Mengqiu Song, Ran Zhao, Hanyong Chen, Kangdong Liu, Jung-Hyun Shim, Feng Zhu, Zigang Dong, Mee-Hyun Lee

**Affiliations:** ^1^China-US (Henan) Hormel Cancer Institute, Zhengzhou, China; ^2^Department of Pathophysiology, School of Basic Medical Sciences, Zhengzhou University, Zhengzhou, China; ^3^The Hormel Institute, University of Minnesota, Minneapolis, MN, United States; ^4^Department of Pharmacy, College of Pharmacy, Mokpo National University, Jeonnam, South Korea; ^5^Cancer Research Institute, The Affiliated Hospital of Guilin Medical University, Guilin, China; ^6^College of Korean Medicine, Dongshin University, Naju, South Korea

**Keywords:** LIMK1, dasatinib, non-small cell lung cancer, off-targets, patient-derived xenografts

## Abstract

Lung cancer is a leading cause cancer-related death with diversity. A promising approach to meet the need for improved cancer treatment is drug repurposing. Dasatinib, a second generation of tyrosine kinase inhibitors (TKIs), is a potent treatment agent for chronic myeloid leukemia (CML) approved by FDA, however, its off-targets and the underlying mechanisms in lung cancer have not been elucidated yet. LIM kinase 1 (LIMK1) is a serine/threonine kinase, which is highly upregulated in human cancers. Herein, we demonstrated that dasatinib dose-dependently blocked lung cancer cell proliferation and repressed LIMK1 activities by directly targeting LIMK1. It was confirmed that knockdown of LIMK1 expression suppressed lung cancer cell proliferation. From the *in silico* screening results, dasatinib may target to LIMK1. Indeed, dasatinib significantly inhibited the LIMK1 activity as evidenced by kinase and binding assay, and computational docking model analysis. Dasatinib inhibited lung cancer cell growth, while induced cell apoptosis as well as cell cycle arrest at the G1 phase. Meanwhile, dasatinib also suppressed the expression of markers relating cell cycle, cyclin D1, D3, and CDK2, and increased the levels of markers involved in cell apoptosis, cleaved caspase-3 and caspase-7 by downregulating phosphorylated LIMK1 (p-LIMK1) and cofilin (p-cofilin). Furthermore, in patient-derived xenografts (PDXs), dasatinib (30 mg/kg) significantly inhibited the growth of tumors in SCID mice which highly expressed LIMK1 without changing the bodyweight. In summary, our results indicate that dasatinib acts as a novel LIMK1 inhibitor to suppress the lung cancer cell proliferation *in vitro* and tumor growth *in vivo*, which suggests evidence for the application of dasatinib in lung cancer therapy.

## Introduction

Internationally, lung cancer continues to be the leading cause of cancer-related deaths among both men and women, causing more than 2.1 million new cases and an estimated 1.8 million deaths annually (Cancer Today – IARC, 2018)^1^. Based on the stage regional differences, 5-year survival of patients with lung cancer ranges from 4 to 17% (American Cancer Society. Cancer facts and figures 2015). The world health organization (WHO) classified lung cancer into two major histologic groups, non-small cell carcinoma (NSCLC) and small cell lung carcinoma (SCLC) (Barta et al., 2019). The myriad risk factors for lung cancer most commonly include lifestyle, environmental and occupational exposures. The roles of these factors play vary depending on geographic location, sex and race characteristics, genetic predisposition, as well as their synergistic interactions (Nasim et al., 2019). As the most common therapies in clinical trials, the combination of surgery, chemotherapy and radiation therapy always could be applied for the treatment of patients with different types and stages of malignancies (Socinski et al., 2013). As acknowledged, cisplatin and other platinum-based drugs could destroy lung cancer cells (Bergethon et al., 2012). However, owing to the shortage of efficient drugs in clinical treatments and their disadvantages in prognosis evaluation, it is desirable to explore new targeted drugs. In epidermal growth factor receptor (EGFR) mutant and anaplastic lymphoma kinase (ALK) rearrangements NSCLC, the ratification and application of EGFR and ALK targeted inhibitors have achieved a pronounced clinical breakthrough, as evidenced by more recently studies for tumors with translocated ROS1 and RET (Drilon et al., 2013). Although EGFR targeted therapy recorded success, it functions in only ∼15% of patients with lung cancer (Aggelou et al., 2018). Consequently, it is urgent to further explore the optimal targets.

The study of subcellular location showed that LIM kinase 1 (LIMK1) is expressed in the whole cell, including cytoplasm and nuclear. LIMK1 overexpression was occurred in various human cancer types such as colorectal cancer and breast cancer (Arber et al., 1998; McConnell et al., 2011; Lee et al., 2019). Phosphorylated LIMK1 (p-LIMK1), which is the active state of LIMK1, is indispensable for cofilin inactivation (phosphorylation of cofilin at Ser3 residue) (Maciver and Hussey, 2002; Manetti, 2012; Su et al., 2016). As for cofilin, it belongs to the actin depolymerization factor (ADF) family and plays an important role in promoting turnover and degradation of actin filaments (Lombardo et al., 2004; Bamburg and Bernstein, 2010; Su et al., 2017). Activation of LIMK1 promotes phosphorylation of ADF/Cofilin, which reduces depolymerization of ADF/Cofilin. It can regulate actin cytoskeletal recombination and further promote the formation and metastasis of filopodia in tumor cells (Su et al., 2017).

Given the high costs, failure rate and long testing periods of developing new medicines, using drugs that are approved for the treatment of diverse diseases as candidate anti-cancer therapeutics represents a faster and cheaper alternative, benefiting from available clinically suitable formulations and evidence of tolerability in patients (Skrott et al., 2017). Dasatinib is widely used in clinical trials as an effective small molecule inhibitor by targeting multiple tyrosine kinases. As a second-generation tyrosine kinase inhibitor (TKI), dasatinib which is 325-fold more potent against inhibition of BCR-ABL has shown advantages in treating newly diagnosed chronic phase chronic myeloid leukemia (CP CML) compared to imatinib. Also, it has responded the earlier and deeper in major molecular response (MMR) and complete cytogenetic response (CCyR). Dasatinib downregulates various tyrosine kinases containing BCR-ABL1 and kinases of SRC family as a FDA approved drug at nanomolar concentration (Kang et al., 2015; Cortes et al., 2017; Scuoppo et al., 2019).

The purpose of current study is to illuminate the effects of dasatinib on the proliferation of lung cancer cells and tumor growth of SCID mice, as well as the relationship between dasatinib and LIMK activity. First, we demonstrate that dasatinib inhibits lung cancer cell proliferation, while induced G1 phase arrest of cell cycle and cell apoptosis *in vitro*. Second, we indicate that dasatinib decreases tumor growth of patient-derived xenograft (PDX) models *in vivo*. Altogether, we unveil that the inhibitory activities of dasatinib against lung cancer cells are realized by regulating LIMK1 activity directly and LIMK1/cofilin signaling pathway.

## Materials and Methods

### Reagents

Dasatinib (purity ≥ 99%) was purchased from Santa Cruz Biotechnology (CAS: 302962-49-8, Lot# H0515) (Beverly, MA, United States). BMS-5 (purity ≥ 98%) was bought from Enzo Life Sciences (CAS:1338247-35-0) (Shanghai, China). Dimethylsulfoxide (DMSO) and CNBr-activated Sepharose^TM^ 4B (Lot# 10265330) were purchased from Sigma (St. Louis, MO, United States) and GE Healthcare (Uppsala, Sweden) respectively. Cyclin D1 (Cat# 2922), cyclin D3 (Cat# 2936), cleaved-PARP (Cat# 5625), caspase-3 (Cat# 9662), cleaved caspase-3 (Cat# 9664) caspase-7 (Cat# 9492), and cleaved caspase-7 (Cat# 8438) were purchased from Cell Signaling Technology (Beverly, MA, United States). p-LIMK (Thr 508/505)-R (Catalog# sc-28409-R), LIMK-1 (Catalog# sc-28370), Cofilin (Catalog# sc-33779), p-Cofilin (mSer3)-R (Catalog# sc-21867-R) were purchased from Santa Cruz Technology. β-actin antibody (Cat# TA-09) as a loading control was obtained from ZSGB-Bio Company (Beijing, China).

### Cell Culture

The human lung cancer cell lines (NCI-H1975 and NCI-H1650) and human bronchial epithelial cells NL-20 were purchased from the American Type Culture Collection (ATCC, Manassas, VA, United States). NL-20 cells were cultured in Ham’s F12 medium, supplemented with 1.5 g/L sodium bicarbonate, 2.7 g/L glucose, 2.0 mM L-glutamine, 0.1 mM non-essential amino acids, 0.005 mg/ml insulin, 10 ng/ml epidermal growth factor, 0.001 mg/ml transferrin, 500 ng/ml hydrocortisone and 4% fetal bovine serum. NCI-H1975 and NCI-H1650 cells were cultured in RPMI-1640 containing penicillin (100 units/ml), streptomycin (100 μg/ml) and 10% fetal bovine serum (Biological Industries, Israel). All cells were grown at 37°C in a humidified incubator containing 5% CO_2_, cytogenetically tested and authenticated before being frozen and stored in liquid nitrogen. The cultured cells were updated by new frozen cells once they were being used less than 2 months.

### Cell Proliferation Assay

Logarithmic phase cells were collected and seeded in each well of 96-well plates at the density of approximately 2 × 10^3^ cells/100 μl, followed with incubation for 24 h prior to treatment with different doses of dasatinib. After 24, 48, and 72 h incubation, 20 μl of MTT [5 mg/ml, 3-(4,5-dimethylthiazol-2-yl)-2,5-diphenyltetrazolium bromide, Ruitaibio, Beijing, China] reagent was added in each well. In order to guarantee the precision of the results, six replicate wells were set for each compound concentration. One hour later, the supernatant was discarded carefully and 100 μl of DMSO was added in each well, when the color of the solution became purple, the absorbance was measured at 570 nm as soon as possible.

### Preparation of LIMK1 Knockdown Cells

For knocking down the expression of LIMK1 in lung cancer cells, 5 μg per well of plasmid, *pLKO.1-mock*, and *shRNA-Limk1* plasmids together with pMD2.0G and psPAX (Addgene Inc., Cambridge, MA, United States), the universal packaging vectors, were first transfected by 30 μl Simple-Fect transfection reagent (Signaling Dawn Biotech, Wuhan, Hubei, China) into 293T cells. After 12 h, the media were changed freshly and cultured for another 48 h. Afterward, NCI-H1650 cells were infected with a mixture of media containing polybrene and shMock or shLIMK1 viral particles, which were filtered with 0.22 μm filter when being harvested. Thereafter, NCI-H1650 cells were treated with puromycin (final concentration of 2 μg per ml) to screen LIMK1 knockdown cells after infection for 24 h. The infected cells were used for further analysis, including anchorage-independent cell growth assay and Western blot analysis.

### Anchorage-Independent Cell Growth Assay

NCI-H1975 and NCI-H1650 cells were suspended in RPMI-1640 complete growth media and cell number was adjusted to 8,000 per well. Afterward, 0.3% agar with different doses of dasatinib was added in a top layer up a base layer of 0.5% agar containing the same corresponding concentrations of dasatinib in 6-well plates, three repeat wells were set for each compound concentration. The cultures were grown at the optimal condition with 37°C and 5% CO_2_. Two or three weeks later, the colonies were photographed under a microscope and counted with the Image-Pro Plus software (v.6.0) program (Media Cybernetics, Rockville, MD) under the same standard.

### Cell Cycle Analysis and Apoptosis Assay

Depending on the growth rate of different cells, a certain number of cells were seeded in 60-mm dishes and cultured overnight, then treated with 0, 2.5, 5, 10, or 20 μM of dasatinib for 24 or 48 h, respectively. For cell cycle analysis, cells were released for 24 h followed by drug treatment, and fixed in cold 70% ethanol overnight at −20°C after cells harvest. After staining with propidium iodide and incubation for 15 min in the dark, flow cytometry (FACSCalibur, BD Biosciences, San Jose, CA, United States) analysis was performed to check the cell cycle distribution. For cell apoptosis assay, cells were released for 48 h and stained with annexin-V (Biolegend, San Diego, CA, United States) for 10 min at the room temperature and stained with propidium iodide (Solarbio, Beijing, China) for 15 min in the dark, and apoptotic cells were measured using flow cytometry (FACSCalibur).

### Western Blot Analysis

After the desired treatment in cell culture, the lung cancer cells were washed with cold PBS and lyzed (50 mM Tris pH 8.0, 0.5–1% NP-40, 150 mM NaCl, protease inhibitor cocktail, and 1 mM PMSF), and then centrifuged at 14,000 rpm for 20 min at 4°C. Protein concentration was quantified by the BCA Quantification Kit (Solarbio, Beijing, China). Equal amounts of cell lysates were separated by the 10–15% SDS-PAGE, and then transferred to polyvinylidene difluoride (PVDF) membranes soaked with methanol in advance. After that, the corresponding PVDF membranes were blocked with 5% milk for 1 h at room temperature, prior to incubation with primary antibodies [all antibodies were used in the system under study (assay and species) according to the instructions of the manufacturer] against p-LIMK (Thr508/505), LIMK1, p-cofilin (Ser3), cofilin, cyclin D1, cyclin D3, CDK2, cleaved PARP, cleaved Caspase 3, cleaved Caspase 7, caspase 3, caspase 7 or β-actin at 4°C overnight. Before following by detection by secondary antibodies: goat anti-mouse IgG–HRP, goat anti-rabbit, the PVDF membranes were washed 3 times with 1 × phosphate buffered saline with 0.05% Tween-20 (PBST) buffer. The target protein bands were visualized by ECL detection reagent (GE Healthcare Life Science, Little Chalfont, United Kingdom) and images were recorded by the Amersham Image 600 (GE, Milwaukee, WI, United States).

### *In vitro* and *ex vivo* Pull-Down Assays

DMSO-sepharose 4B and dasatinib-sepharose 4B beads were mixed with 500 μg cell lysates respectively, a certain volume of reaction buffer (50 mM Tris pH 7.5, 5 mM EDTA, 150 mM NaCl, 1 mM DTT, 0.01% NP-40, and 2 mg/ml bovine serum albumin) was added up to a final volume of 500 μl, and then incubated with gentle rocking at 4°C overnight. After centrifugation at 12,500 rpm for 1 min, the samples were washed 5 times with washing buffer. The washing buffer shared almost the same compositions with the reaction buffer except for the concentration of bovine serum albumin. The positive results were visualized by Western blotting.

### Computational Docking Model

In order to investigate whether dasatinib can bind with LIMK1 or not, the Schrödinger Suite 2016 software programs were used to complete the docking model (Schrödinger, 2016). The structure of LIMK1 was built with Prime that refined and minimized loops in the interacting and binding sites. The binding structure between dasatinib and LIMK1 was made according to the standard methods of the Protein Preparation Wizard (Schrödinger Suite 2016). Hydrogen atoms were added to maintain the pH at 7 and all water molecules were discarded. The LIMK1 ATP-binding site under the receptor grid was produced for docking. Hence, we could obtain dasatininb computational docking site.

### Patient Derived Xenograft Mouse Model

Patient-derived tumor (LG52) was cut into the same mass and then implanted subcutaneously into the back of the female SCID mice which were about 6–8 weeks old. Current study was approved by the Ethics Committee of Zhengzhou University (Zhengzhou, Henan, China). At 1 week after tumor implantation, the tumor volume needs to be monitored, the mice were randomly divided into two groups once the average tumor volume reached about 100 mm^3^, (1) vehicle group (*n* = 8); (2) 30 mg/kg dose of dasatinib group (*n* = 8). Dasatinib and vehicle were administered by gavage daily for 36 days, and the tumor volumes were measured twice in a week. Tumor volume was calculated according to the following equation: (mm^3^) = (length × width × height × 0.52). Tumors were isolated from mice and weighed before the average volume exceeding about 1,000 mm^3^.

### Immunohistochemistry (IHC) Assay

Paraffin-embedded tumor tissues were prepared for H&E staining and IHC analysis. When antigen retrieval finished, the tumor tissues were treated with H_2_O_2_ for 5 min and blocked with 5% goat serum, then incubated with primary antibodies to detect the expression of target protein markers, such as Ki-67, p-Limk1/2 (Thr508/505), and p-cofilin (Ser3) at 4°C overnight. After incubation with the proper secondary antibodies, DAB (3,3′-diaminobenzidine) staining was used to visualize the target proteins. Hematoxylin and alcohol were used to counterstained the sectioned tissues and dehydrated, respectively. The stained tissues were photographed under a microscope and analyzed using the Image-Pro Plus software (v.6.0) program (MediaCybernetics, Rockville, MD, United States).

### Statistical Analysis

Data illustrated with error bars were mean ± *SD*. All statistical tests performed were two-tailed independent *t*-test. For all analyses, *p*-value less than 0.05 was considered as statistically significant difference. Each experiment was replicated for three times.

## Results

### Dasatinib Directly Inhibits LIMK1 Kinase Activity

**FIGURE 1:**
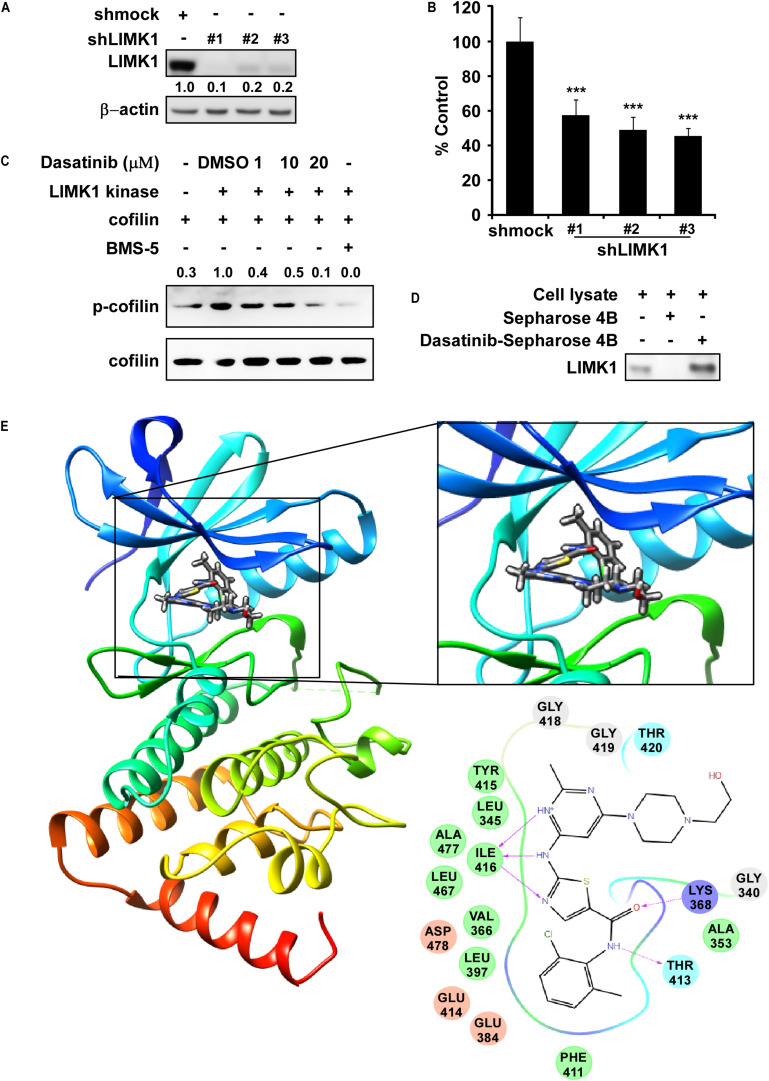
LIMK1 is a target of Dasatinib. **(A)** The expression of LIMK1 after knocking-down LIMK. **(B)** Anchorage-independent colony formation in shmock- or shLIMK infected cells. ***, statistical significance (*p* < 0.001) compared to shmock control. **(C)** Dasatinib inhibits LIMK1 activity and downregulates p-cofilin. **(D)** Dasatinib binds with LIMK1. **(E)** Dasatinib binds with LIMK1 (left); enlarged view of the binding location (right upper); interactions with amino acid sites (right down). LIMK1 structures are presented as ribbon representation and dasatinib is displayed in stick representation with hydrogen bonds.

To verify the role of LIMK1 in lung cancer cells, we first infected shMock or shLIMK #1, #2, and #3 to NCI-H1650 cells, and results of western blot exhibited that LIMK1 expression was lowered by shLIMK in comparison with shMock (Figure 1A). Thereafter, results of anchorage-independent colony growth showed that the colony number of shLIMK contained cells was decreased compared to shMock infected cells (Figure 1B). To investigate the LIMK1 inhibitor, we screened FDA approved drugs by the target based *in silico* computational docking and found dasatinib as a novel LIMK1 inhibitor candidate. For verifying the effect of dasatinib against LIMK, we implemented an *in vitro* kinase assay with active LIMK1 at the presence of 1, 10 or 20 μM of dasatinib and results showed that p-cofilin, a LIMK1 substrate, was inhibited by treatment with dasatinib in a dose dependent manner compared with control group (Figure 1C). We then conducted an *ex vivo* pull-down assay with H1975 cell lysate and found that dasatinib directly bound with LIMK1 (Figure 1D). To illustrate how dasatinib interacted with LIMK1, we created a computational model by docking dasatinib against LIMK1. The results revealed that dasatinib formed hydrogen bonds at lysine (LYS) 368, threonine (THR) 413, and isoleucine (ILE) 416 in the backbone of LIMK1 (Figure 1E). Taken together, these results indicated that dasatinib might be a potential effective inhibitor of LIMK1.

### Dasatinib Inhibits the Proliferation of Lung Cancer Cells

**FIGURE 2:**
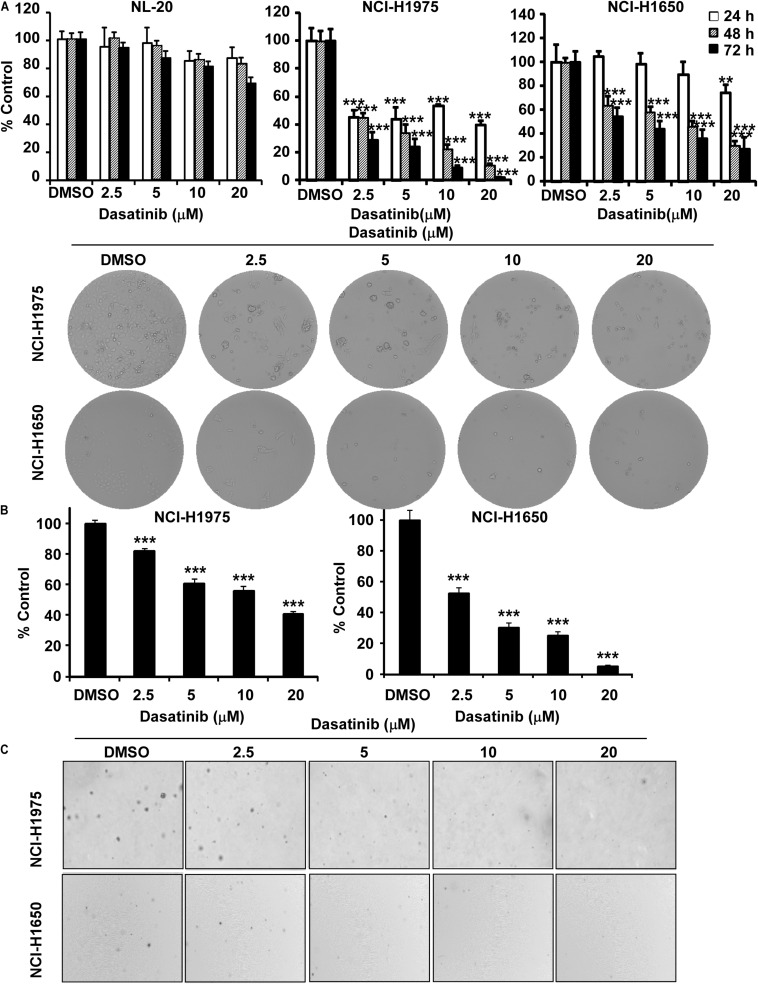
Dasatinib inhibits lung cancer cells growth. **(A)** The effect of dasatinib on the proliferation of normal cells, NCI-H1975 and NCI-H1650 lung cancer cells. Cell proliferation is detected by MTT assay. Cell morphology changes of NCI-H1975 and NCI-H1650 cells after dasatinib treatment were observed by optical microscopy (×100). **(B)** The effect of dasatinib on anchorage-independent cell growth. **(C)** Representative photographs of anchorage-independent cell growth assay. Data are shown compared with DMSO treated cells. **, ***, statistical significance (*p* < 0.01, *p* < 0.001) compared to controls.

In order to assess the value of dasatinib regarding therapeutic dose and toxicity against normal cells, we first investigated the effect of dasatinib on the proliferation of human bronchial epithelial cells (NL20). MTT assay results showed that, after incubation of NL20 cells with dasatinib (2.5, 5, 10 or 20 μM) for 24, 48, and 72 h, even the highest concentration and longest incubation of dasatinib exerted not much toxicity (Figure 2A). Therefore, we selected the maximal non-toxic concentration (20 μM) of dasatinib for further experiments. Dasatinib treatment (5, 10 or 20 μM) significantly inhibited NCI-H1975 and NCI-H1650 lung cancer cell proliferation in a time and concentration-dependent manner, and the IC_50_ values were 0.95 μM and 3.64 μM, respectively at 72 h (Figure 2A). After 72 h incubation with dasatinib (2.5, 5, 10, or 20 μM), the cells lost their spindle-shaped form and became less irregular in structure, cellular debris, membrane became rounded and further shrinking of nuclei compared to untreated cells. Dasatinib also attenuated anchorage-independent growth of these two lung cancer cells in a concentration-dependent manner compared to the DMSO treated control (Figure 2B). Representative images of colonies illustrated the number of colonies (Figure 2C).

### Dasatinib Induces Cell Cycle Arrest at G1 Phase in Lung Cancer Cells

**FIGURE 3:**
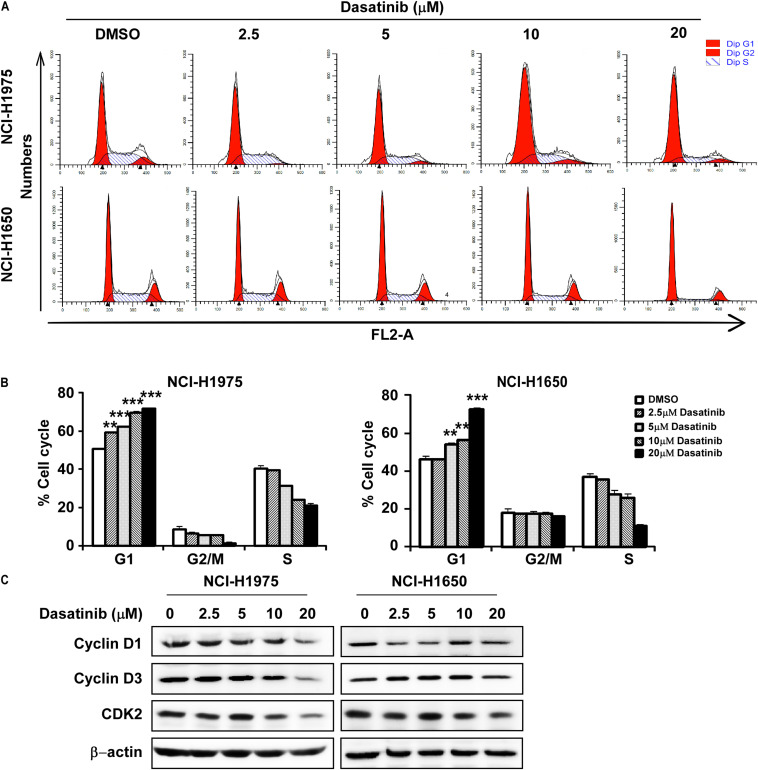
Dasatinib induces cell cycle arrest at G1 phase **(A)** Dasatinib induced cell cycle arrest at G1 phase. **(B)** Dasatinib induces cell cycle arrest at G1 phase in a concentration dependent manner. **(C)** Western blotting results showed the decreased expression of cyclinD1, cyclinD3, and CDK2, G1 phase marker for cell cycle. The asterisks (*p < 0.05, **p < 0.01, ***p < 0.001) indicated a significant difference between untreated control and dasatinib-treated cells. Data were shown as means ± *SD* of values from triplicate samples and similar results were obtained from three independent experiments.

Then we evaluated the role of dasatinib on cell cycle. Treatment with dasatinib for 24 h caused G1 phase cell cycle arrest in NCI-H1975 and NCI-H1650 cells (Figures 3A,B). The result was largely consistent with decreased expressions of cyclin D1, D3, and CDK2, which are typical cell cycle markers at G1 phase (Figure 3C).

### Dasatinib Induces Cell Apoptosis in Lung Cancer Cells

**FIGURE 4:**
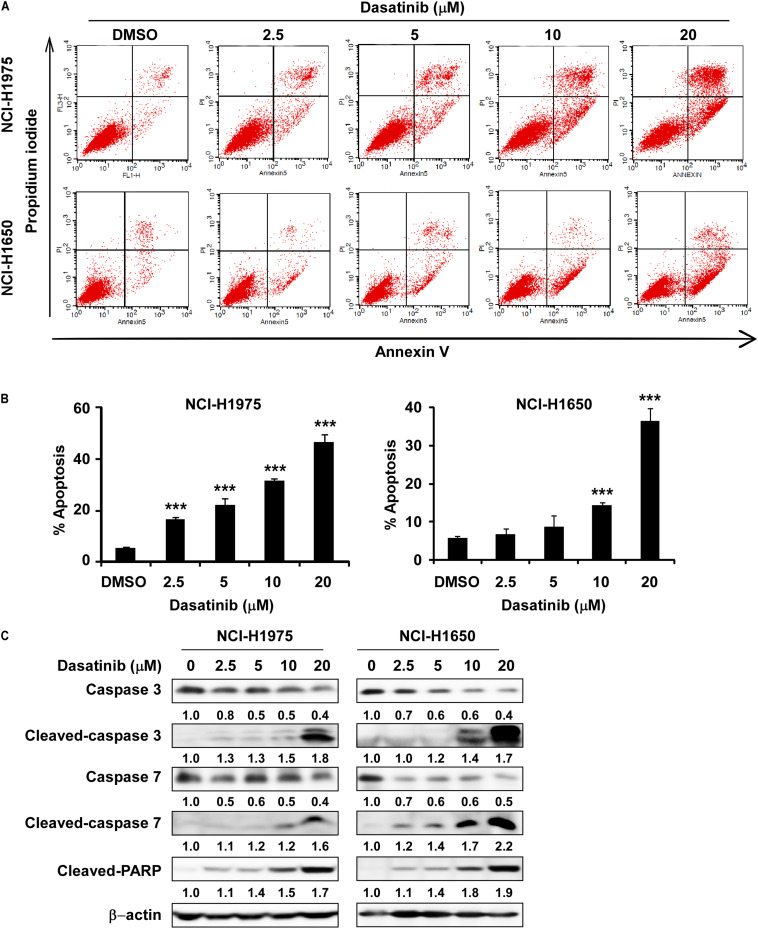
Dasatinib induces cell apoptosis. **(A)** Dasatinib treatment induced cell apoptosis. **(B)** Dasatinib treatment can induce cell apoptosis in a concentration dependent manner. **(C)** Dasatinib upregulated the expression of cell apoptosis markers like cleaved-PARP, cleaved-caspase3 and 7 by determined with Western blotting. The asterisks (*p < 0.05, **p < 0.01, ***p < 0.001) indicated a significant difference between untreated control and dasatinib-treated cells. Data were shown as means ± *SD* of values from triplicate samples and similar results were obtained from three independent experiments.

In the next, we proved that dasatinib induced apoptosis in NCI-H1975 and NCI-H1650 cells at 10 and 20 μM (Figures 4A,B). In order to verify the conclusion, we performed Western blot using the whole-cell lysate treatment with different concentration of dasatinib on the expression of apoptosis biomarkers. The level of cleaved caspase 3, cleaved caspase 7 and cleaved PARP were increased, while the caspase-3 and caspase-7 expression significantly decreased in a concentration-dependent manner (Figure 4C).

### Dasatinib Regulates LIMK1 Downstream Signaling Pathways

**FIGURE 5:**
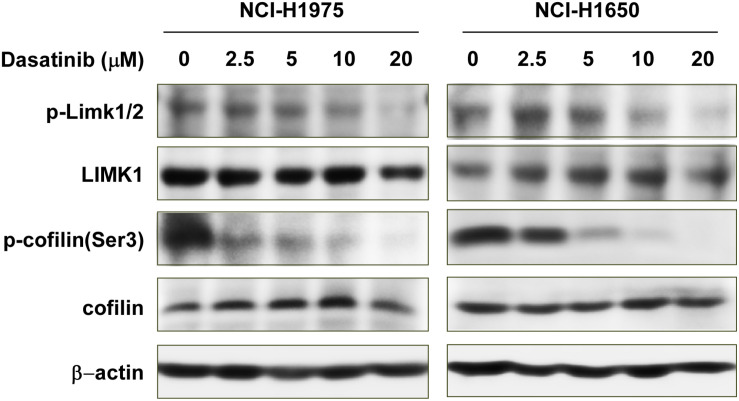
Dasatinib inactivates LIMK1-related signaling pathways. Treatment with dasatinib downregulates p-LIMK (Thr 508/505) and p-cofilin.

Because LIMK1 is a target of dasatinib, we evaluated the effects of dasatinib treatment on LIMK1 downstream signaling pathways. Cofilin is the downstream target protein of LIMK, which can be phosphorylated by LIMK and dephosphorylated by slingshot phosphatases at Serine 3. Cofilin phospho-regulation is crucial for tumor regression (Scuoppo et al., 2019). phosphorylation of cofilin was diminished with 5, 10, or 20 μM dasatinib treatment compared to vehicle-treated cells (Figure 5). However, there were no significant changes in the expression of LIMK1 or cofilin total forms (Figure 5).

### Dasatinib Induces LIMK-Mediated Anti-tumor Efficacy *in vivo*

**FIGURE 6:**
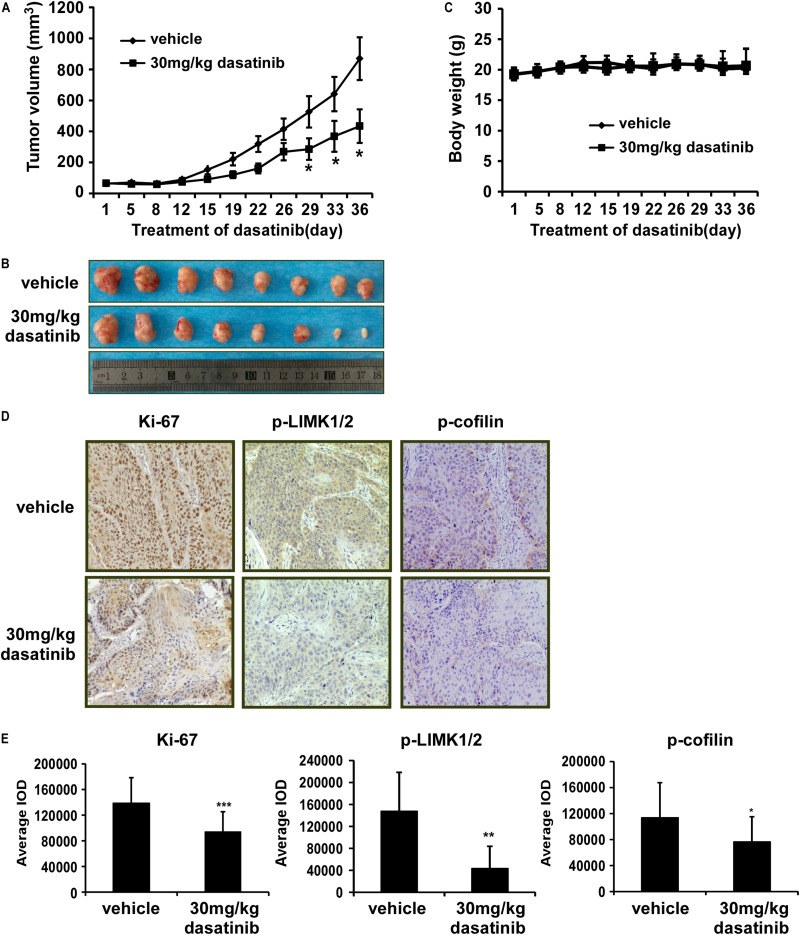
Dasatinib attenuates the growth of PDX tumors in mice. **(A)** The effect of dasatinib on the volume of PDX tumors is plotted over 36 days. Vehicle or 30 mg/kg dasatinib is administered by gavage. Tumor volume is measured twice a week. The asterisk (*p < 0.05) indicates a significant decrease in volume of tumors from dasatinib-treated mice in comparison with vehicle-treated mice. Data are shown as mean ± *SD* values. **(B)** The photographs of tumors from PDX mice treated with vehicle or 30 mg/kg dasatinib. **(C)** Body weight of mice is plotted over 36 days. **(D)** The expression of Ki-67, p-LIMK, and p-cofilin is examined by IHC analysis (100X magnification). **(E)** The expression of Ki-67, p-LIMK and p-cofilin are quantified from 4 separate areas on each slide. Data are expressed as IOD values ± *SD*. The asterisks (*p < 0.05, **p < 0.01, ***p < 0.001) indicate a significant decrease in Ki-67, p-LIMK, and p-cofilin in dasatinib treated tissues compared to vehicle treated controls.

Thereafter, in order to further explore the anti-tumor effects of dasatinib *in vivo*, we used PDX tumor model in SCID mice. Results showed that dasatinib significantly inhibited tumor growth compared to vehicle group without loss of body weight (Figures 6A–C). Then we examined the expression of Ki67, p-LIMK1/2, and p-cofilin in xenograft tumor sections using the IHC analysis. Dasatinib remarkably inhibited the expression of Ki67, p-LIMK1/2, and p-cofilin protein in tumor tissues from dasatinib-treated group as compared to vehicle treated group (Figures 6D,E). Above findings demonstrated that the anti-tumor activity of dasatinib was mediated by LIMK1 in PDX mice model.

## Discussion

Nowadays, lung cancer is a leading cause of death in both men and women worldwide. There is no clear explanation for its mortality rate (Romaszko and Doboszynska, 2018). Whereas, we know that both genetic and environmental factors influence cancer therapy. Although there are many FDA-approved treatments, such as growth factor receptor inhibitors and immunotherapy currently in clinical use, these treatments may not be fully effective for all patient populations. For research scientists, therefore, the search for new treatments remains an ongoing adventure. Dasatinib, a tyrosine kinase inhibitor, markedly reduces tyrosine phosphorylation of p130cas, paxillin, and vinculin, which are localized to the actin cytoskeleton in the focal adhesions. p130cas, a prominent Src substrate, is a major component of focal adhesion and it acts as a mechanosensor of force (Janostiak et al., 2014). It has been reported that dasatinib inhibits the migration and metastasis of canine mammary cancer cells with enhanced Wnt and HER signaling via downregulation of cSRC (Timmermans-Sprang et al., 2019), and inhibits actin fiber reorganization and promotes endothelial cell permeability through RhoA-ROCK Pathway (Dasgupta et al., 2017; Lee et al., 2019). Dasatinib also inhibited the invasion of lung cancer cells (A549 and H1299) contained EGFR wildtype and gefitinib-resistance (H1975) contained EGFR mutation (Song et al., 2006). In clinical study, the treatment of dasatinib combined with erlotinib in 13 advanced NSCLC patients for phase I/II showed two partial responses and one bone response (Haura et al., 2010). Among the 34 advanced NSCLC patients, single treatment of dasatinib showed 11 metabolic responses and one partial response (Johnson et al., 2010). Nevertheless, the effects of dasatinib in lung cancer were yet to be fully elucidated. In this study, we have found that treatment of NCI-H1975 and NCI-H1650 cells with dasatinib resulted in significant inhibition of anchorage independent colony formation, and induction of G1 phase cell cycle arrest and apoptosis (Figures 2–4). The changes in the expression of apoptosis markers such as increased PARP cleavage, cleaved-caspase3 and 7 further supported the anti-tumor role of dasatinib in lung cancer cells. We further explored LIMK1 as a new target of dasatinib, which directly binds with LIMK1 (Figure 1C). Moreover, shRNA-mediated knock down of LIMK1 in NCI-H1650 cells reduced the colony formation. These findings uncovered dasatinib is a potential inhibitor for lung cancer, and LIMK1 is a new target of dasatinib. As known, ROCK1/LIMK1/COFILIN1 pathway regulates actin cytoskeletal dynamics and enhances migration (McConnell et al., 2011; Chen et al., 2013). Actin, as a highly abundant cytoskeletal, extensively exits in eukaryotic cells, plays a significant role in modulating various structural and functional roles. The polymerization and depolymerization of actin filaments are controlled by actin binding protein. LIMK kinase phosphorylates and inactivates cofilin, which is a low molecular weight actin-binding protein. Actin nuclear transport, actin cytoskeleton re-organization and cytokinesis are related to phosphorylated cofilin protein. The actin and microtubule cytoskeletons are critically important for cancer cell proliferation, and drugs that target microtubules are widely used cancer therapies (Yoshioka et al., 2003). Our result further showed that dasatinib decreased the protein expressions of p-LIMK1/2 and p-cofilin (Figures 5, 6).

PDX models are ideal tumor animal models. The tumors derived from patients with lung cancer were implanted into the SCID mice, which maintained the heterogeneity and biological characteristics of tumor (Kopetz et al., 2012). Therefore, we performed the PDX models as an essential part to evaluate the anti-tumor effect of dasatinib *in vivo*. Compared with vehicle group, the tumor volume in the dasatinib-treated mice were significantly decreased (Figures 6A–C). We also checked the expression of Ki67, p-LIMK1/2 and downstream p-cofilin in the tumors by using IHC assay and three markers are all downregulated in the dasatinib-treated tumors compared to vehicle group (Figures 6D,E). From our studies, PDX tumors exhibiting higher p-LIMK1 expression showed more dramatic decrease in tumor volume as well as related IHC biomarker staining when treated with dasatinib.

In the current study, we checked the efficiency of dasatinib in *in vitro* and *in vivo* studies. For *in vitro* experiments, dasatinib inhibited LIMK activity by negatively regulating the phosphorylation of the LIMK1 substrate, cofilin, which resulted in growth inhibition, apoptosis and cell cycle arrest in lung cancer cell lines that were highly expressed LIMK1. For *in vivo* experiments, the tumor progression was significantly retarded in dasatinib-treated group compared with vehicle group. The therapeutic effects of dasatinib also exhibit a positive relationship with the expression level of LIMK. LIMK can be regulated by gene expression via microRNAs such as miR-320a, miR-138, and miR-27b (Wan et al., 2014; Tan et al., 2016; Qin et al., 2020). Current study provides a message useful for moving dasatinib toward the treatment for patients with lung cancer and shows the prospective translational potential of dasatinib for patients with lung cancer who were accompanied with high expression of LIMK1. More broadly, our study illustrates a potential drug reuse approach, provides novel insights, identifies new lung cancer related targets and encourages further clinical trials. In any event, the use of dasatinib in lung cancer therapy should be considered as a possibility, and it should be recognized that more studies are required.

## Data Availability Statement

The raw data supporting the conclusions of this article will be made available by the authors, without undue reservation, to any qualified researcher.

## Ethics Statement

The animal study was reviewed and approved by the Ethics Committee of Zhengzhou University (Zhengzhou, Henan, China).

## Author Contributions

MZ, RW, JT, M-HL, and ZD were involved in study concept and design, acquisition of data, analysis and interpretation of data, drafting of the manuscript. MZ, RW, and JT performed the experiments. MS, RZ, KL, J-HS, FZ, and HC supported the data analysis and materials. MZ, RW, M-HL, and ZD wrote the manuscript. ZD and M-HL had supervision of all study. All authors read and approved the final manuscript.

## Conflict of Interest

The authors declare that the research was conducted in the absence of any commercial or financial relationships that could be construed as a potential conflict of interest.

## Footnotes

1 https://gco.iarc.fr/today/data/factsheets/cancers/15-Lung-fact-sheet.pdf
